# Supplementary material for: Systematic comparison of respiratory syncytial virus-induced memory B cell responses in two anatomical compartments
Source: Nat Commun. 2019 Mar 8;10:1126. doi: 10.1038/s41467-019-09085-1 (PMC6408481; doi:10.1038/s41467-019-09085-1)
Supplement: Supplementary file 1 — Supplementary Information [file 41467_2019_9085_MOESM1_ESM.docx]

Supplementary Information

**Systematic comparison of respiratory syncytial virus-induced memory B cell responses in two anatomical compartments**

Shehata *et al.*

**
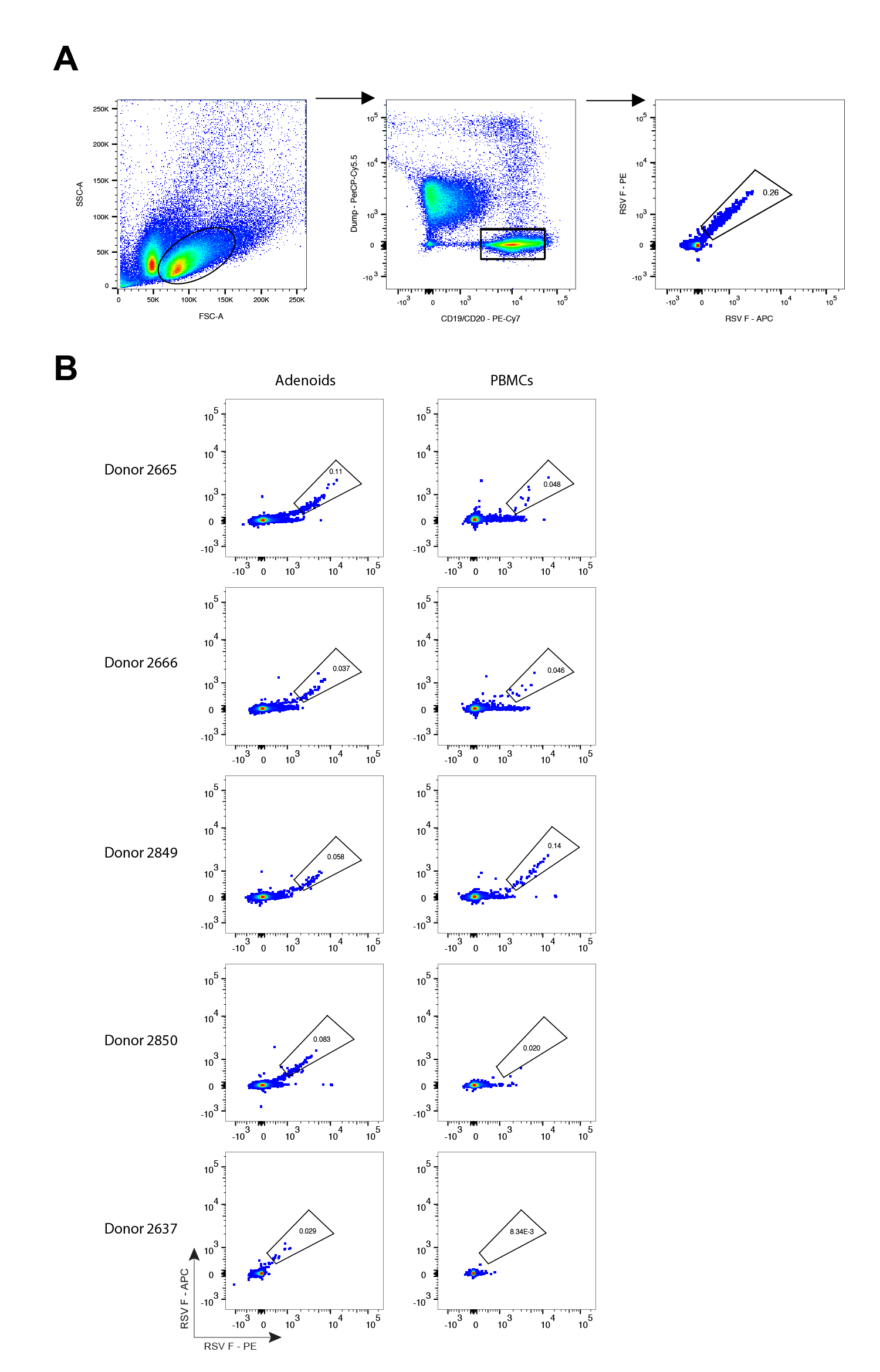
**

**Supplementary Figure 1. RSV F-specific B cell sorting.** (**a)** Representative fluorescence activated cell sorting (FACS) gating strategy used for RSV F-specific B cell sorting. Lymphocytes were gated based on forward and side scatter, followed by a live/dead gate and selection of CD3^−^CD8^−^CD14^−^ cells. B cells were identified by gating on CD19^+^/CD20^+^ cells. B cells that showed reactivity with RSV F were single-cell sorted for antibody cloning. (**b**) Shown is the frequency of RSV F-reactive B cells among CD19^+^ B cells in adenoid (left) and PBMCs (right) for all donors except Donor 2635 (Donor 2635 plots are shown in Figure 1A). SSC-A, side scatter area; FSC-A, forward scatter area. RSV F was labeled with two different fluorophores to reduce background binding.

**
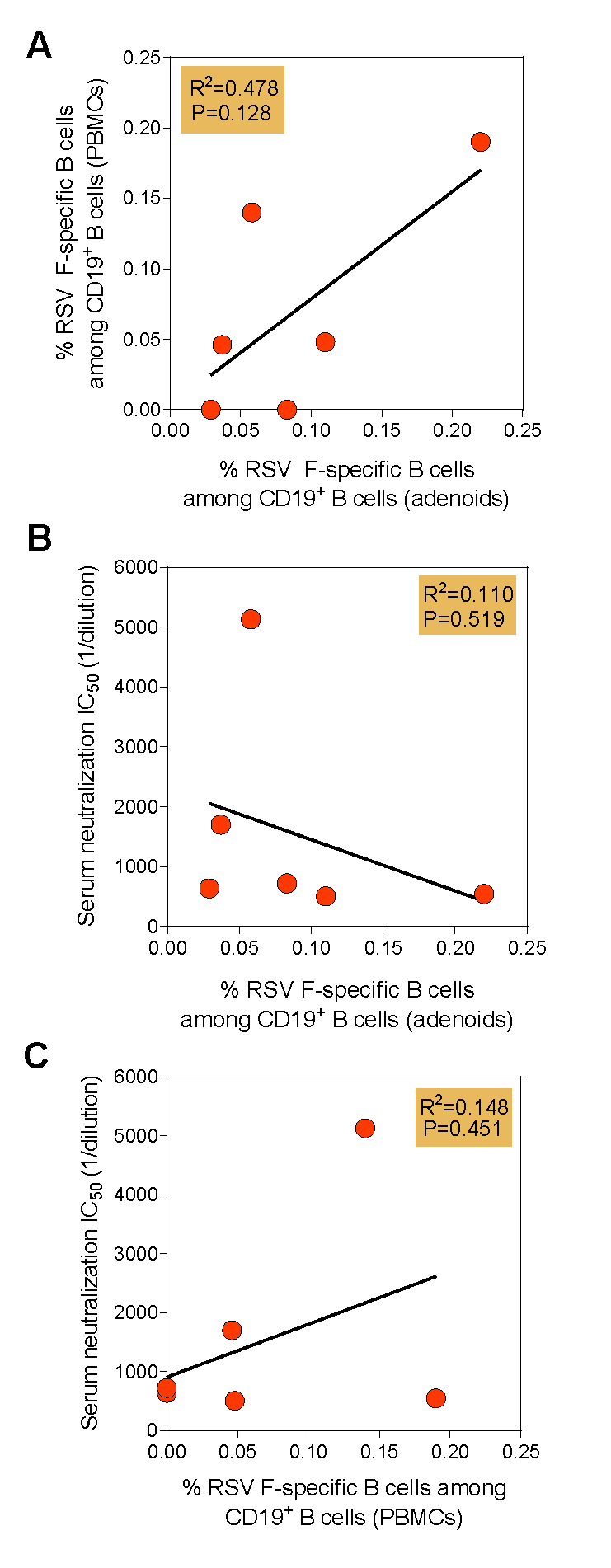
**

**Supplementary Figure 2. Comparison of antibody responses in adenoids and PBMCs.** (**a**) Correlation between the frequency of RSV F-reactive memory B cells in adenoids and PBMCs. (**b)** Correlation between plasma neutralization and frequency of RSV F-reactive memory B cells in adenoids. (**c**) Correlation between plasma neutralization and frequency of RSV F-reactive memory B cells in peripheral blood.

**
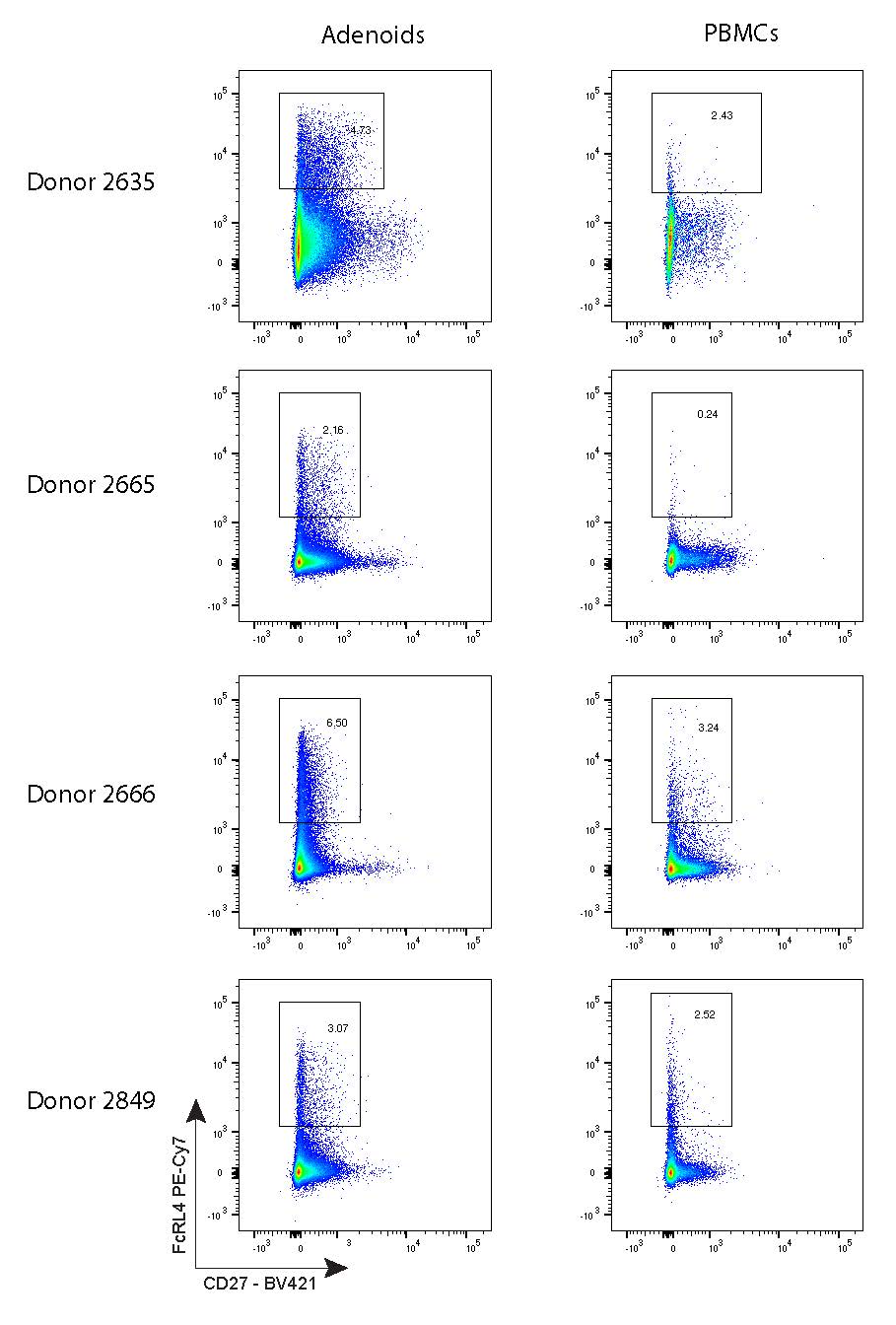
**

**Supplementary Figure 3. FcRL4 expression on B cells in adenoids and PBMCs.** Flow cytometric analysis of FcRL4 expression on B cells in adenoids and PBMCs. Plots shown are gated for CD19/CD20^+^ B cells with the FcRL4^+^ B cells marked by a box. The frequency of FcRL4^+^ B cells within the CD19/CD20^+^ B cell population is shown inside gate.

**
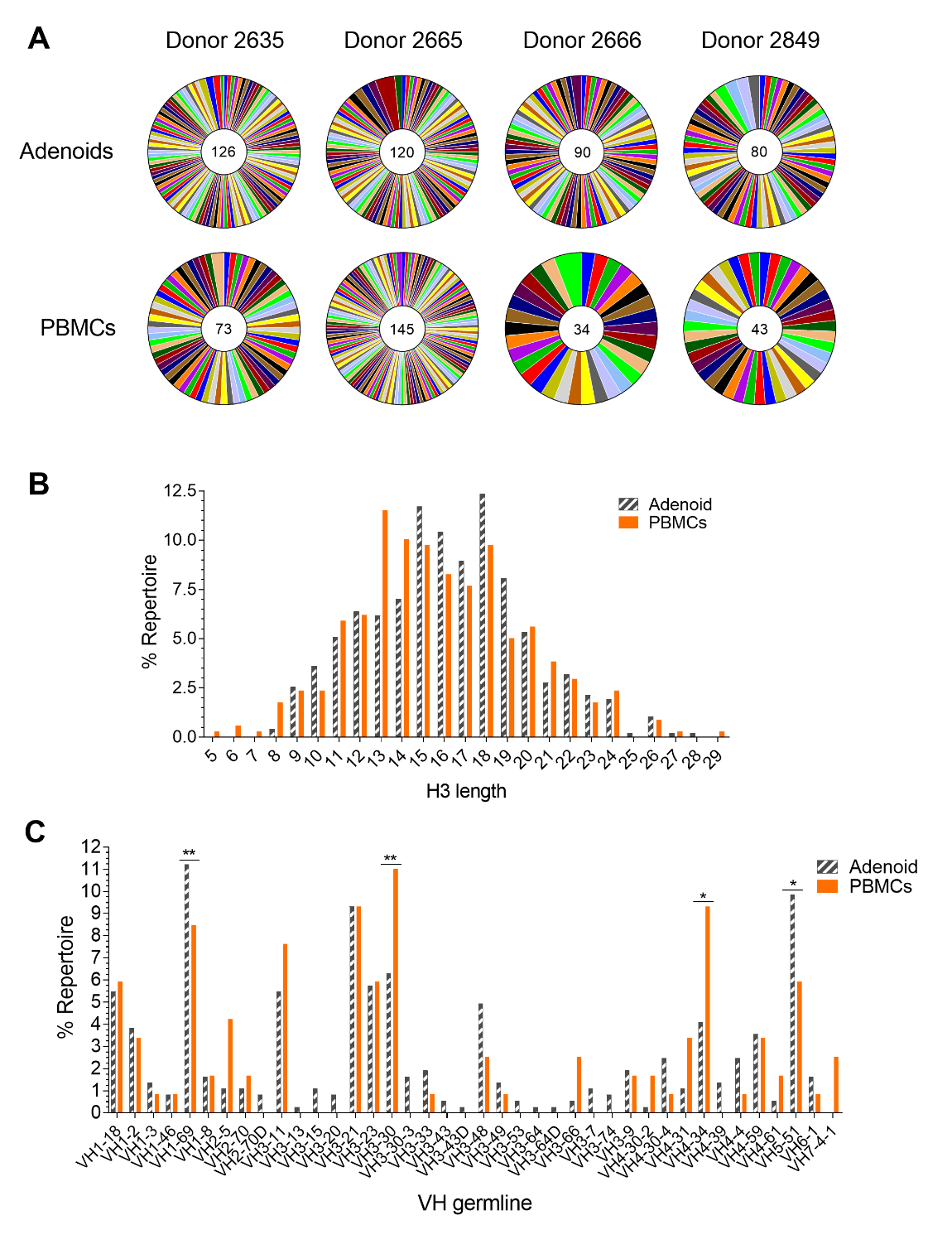
**

**Supplementary Figure 4.** **Sequence analysis of RSV F-specific antibodies.** (**a**) Number of clonal lineages isolated from adenoids and PBMCs from each donor. Each slice of the pie represents one clonal lineage and the size of the slice is proportional to the number of clones in each lineage. The number in the center of the pie denotes the total number of antibodies. (**b**) CDRH3 length distribution of antibodies isolated from adenoids and PBMCs. (**c**) VH germline gene usage of antibodies isolated from adenoids and PBMCs. Antibodies from all donors were pooled for the analysis of CDRH3 length distribution and VH germline gene usage. Statistical comparisons were made using Fisher’s exact test (** P < 0.01, * P < 0.05)


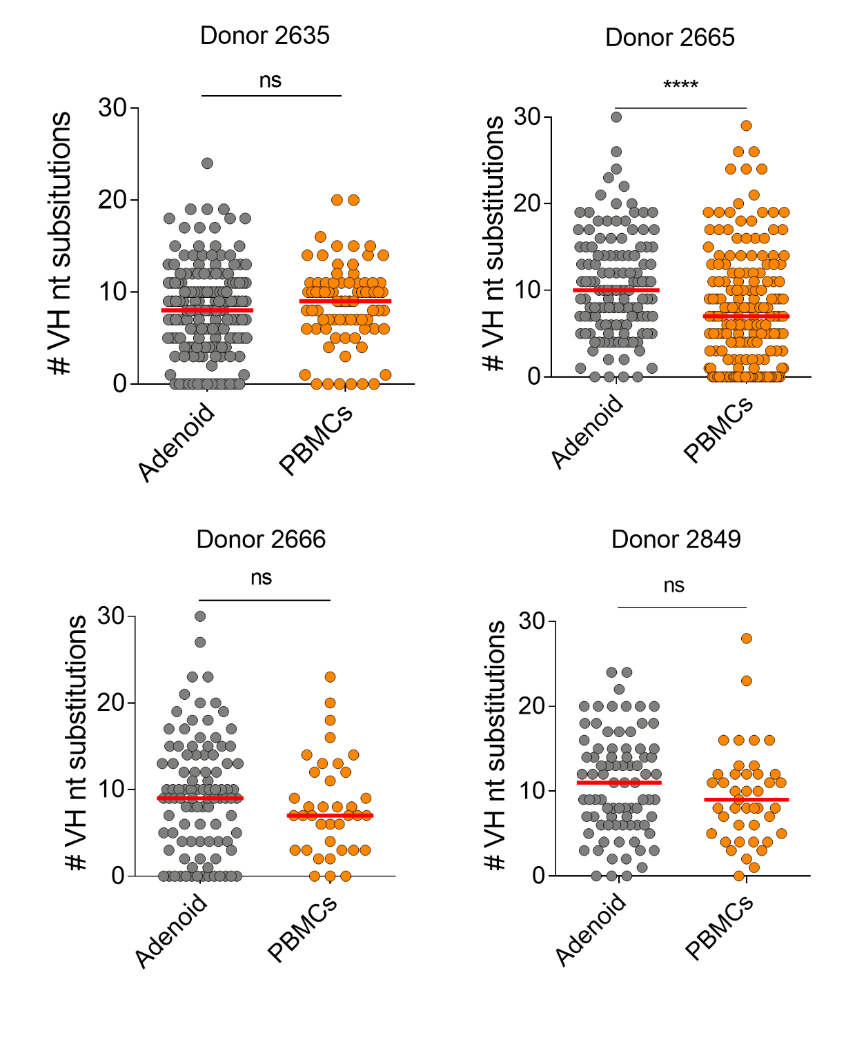


**Supplementary Figure 5.** **Somatic hypermutation load in adenoid- and PBMC-derived antibodies.** Load of somatic mutations (expressed as the number of nucleotide substitutions in VH) in antibodies isolated from adenoids and PBMCs from each donor. Each point represents an individual antibody. Red bars indicate the median number of nucleotide substitutions. Statistical comparisons were made using the Mann-Whitney test (**** P <0.0001, n.s. = not significant).


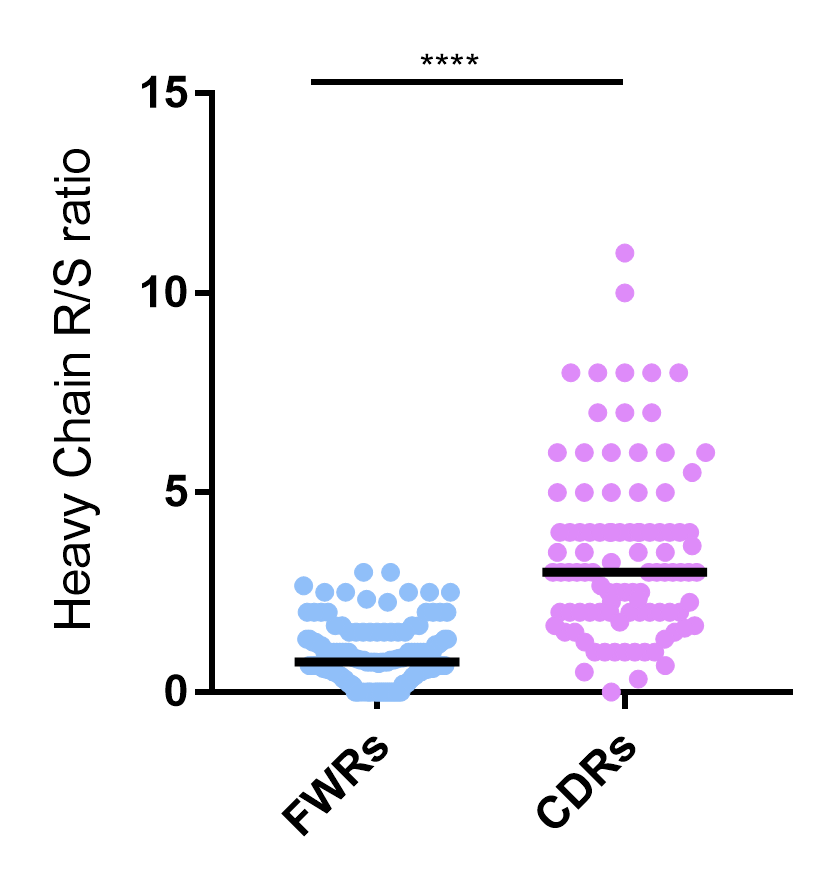


**Supplementary Figure 6. Replacement (R) and silent (S) mutation ratio in FWR and CDR regions of antibodies derived** **from RSV F-reactive, IgG^—^IgA^—^CD27^—^ adenoid B cells.**  Shown is the ratio of replacement to silent nucleotide exchanges in the VH FWRs 1-3 and CDRs 1-2 in antibodies derived from RSV F-specific IgG^—^IgA^—^CD27^—^ B cells (*n* = 149). Statistical comparisons were made using the Mann-Whitney test (*** P <0.001). Black bars indicate medians.

**
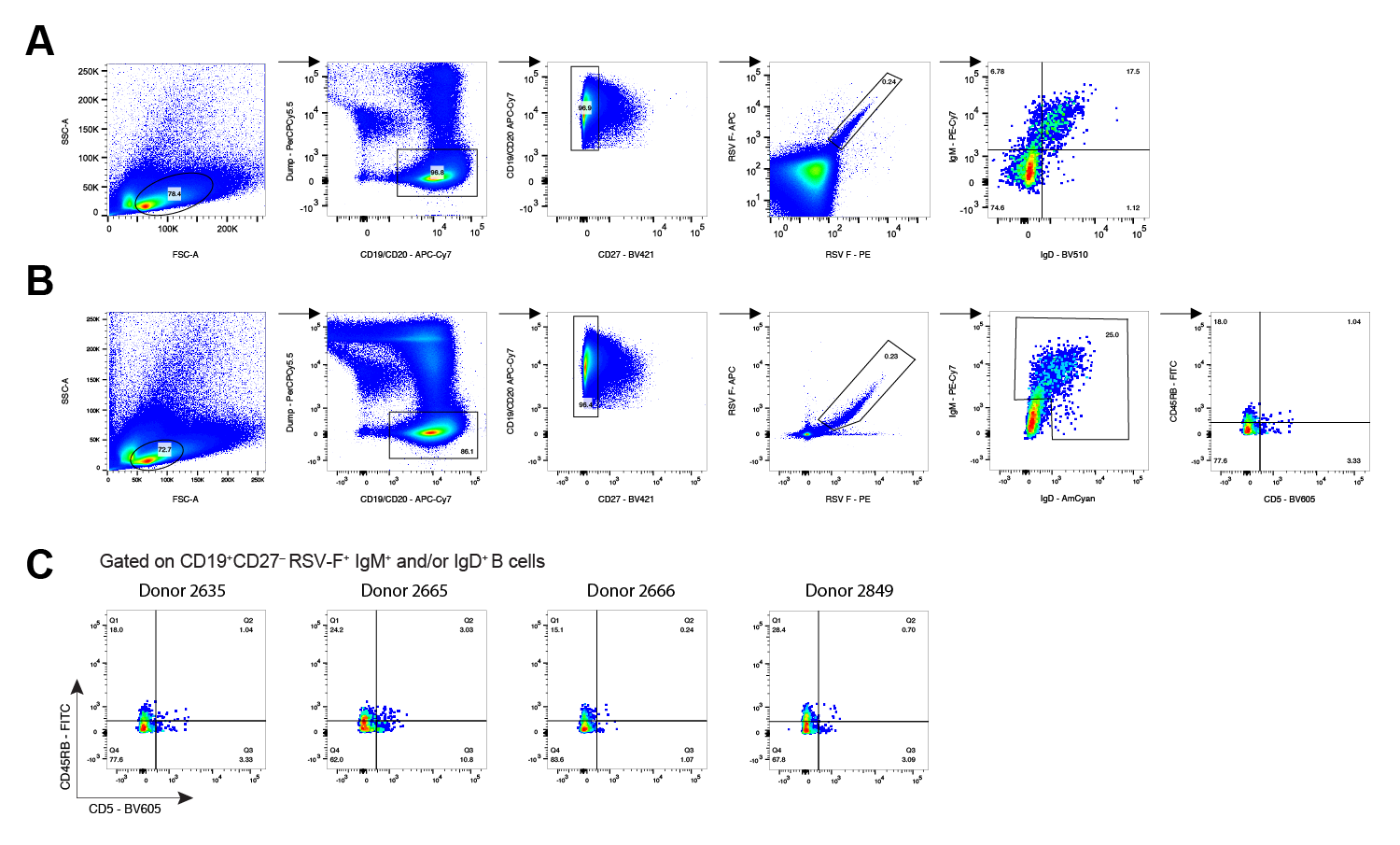
**

**Supplementary Figure 7.** **IgM, IgD, CD5, and CD45RB staining.** (**a**) Representative FACS gating strategy used for determining the percentage of RSV F-reactive, CD27**^—^** adenoid B cells that express IgM and/or IgD. (**b**) Representative FACS gating strategy used for determining the percentage of CD19^+^CD27**^—^**RSV F^+^ IgM^+^/IgD^+^ adenoid B cells that express CD5 and/or CD45RB^MEM55^. (**c)** Percentages of CD19^+^CD27**^—^**RSV F^+^ IgM^+^/IgD^+^ adenoid B cells that express CD5 and/or CD45RB^MEM55^ are shown for all 4 donors. Gating was performed as in (**b**). SSC, side scatter area; FSC, forward scatter area. RSV F was labeled with two different fluorophores to reduce background binding.

**Supplementary Table 1. Donor characteristics.**

|  |  |  |  | **50% RSV neutralization titer (1/dilution)** | | |
| --- | --- | --- | --- | --- | --- | --- |
| **Donor** | **Gender** | **Age (yrs)** | **Surgical Indication** | **Plasma** | **Adenoid filter** | **Adenoid supernatant** |
| 2635 | M | 3.60 | apnea/obstructed breathing | 545 | 9 | 9 |
| 2637 | F | 3.13 | recurrent otititis | 639 | <4 | 13 |
| 2665 | M | 2.82 | apnea/obstructed breathing and recurrent otititis | 506 | <4 | <4 |
| 2666 | F | 3.05 | apnea/obstructed breathing | 1702 | 13 | 177 |
| 2849 | M | 2.79 | recurrent otititis | 5133 | 4 | 55 |
| 2850 | M | 2.97 | apnea/obstructed breathing and recurrent otititis | 720 | 6 | 5 |
