# Supplementary material for: Systematic comparison of respiratory syncytial virus-induced memory B cell responses in two anatomical compartments
Source: Nat Commun. 2019 Mar 8;10:1126. doi: 10.1038/s41467-019-09085-1 (PMC6408481; doi:10.1038/s41467-019-09085-1)
Supplement: Supplementary file 3 — Description of Additional Supplementary Files [file 41467_2019_9085_MOESM3_ESM.pdf]

**Description of Additional Supplemenary Files**

**File:** Supplementary Data 1
**Descrition:** Binding, neutralization, and sequence properties of anti-RSV antibodies

**File:** Supplementary Data 2
**Descrition:** Primer sequences for single B cell cloning
