# Supplementary material for: Systematic comparison of respiratory syncytial virus-induced memory B cell responses in two anatomical compartments
Source: Nat Commun. 2019 Mar 8;10:1126. doi: 10.1038/s41467-019-09085-1 (PMC6408481; doi:10.1038/s41467-019-09085-1)
Supplement: Supplementary file 6 — Reporting Summary [file 41467_2019_9085_MOESM6_ESM.pdf]

nature research

Corresponding author(s): NCOMMS-18-35590

Reporting Summary

Nature Research wishes to improve the reproducibility of the work that we publish. This form provides structure for consistency and transparency in reporting. For further information on Nature Research policies, see Authors & Referees and the Editorial Policy Checklist.

Statistical parameters

When statistical analyses are reported, confirm that the following items are present in the relevant location (e.g. figure legend, table legend, main text, or Methods section).

n/a	Confirmed	
☐	☒	The exact sample size (*n*) for each experimental group/condition, given as a discrete number and unit of measurement
☒	☐	An indication of whether measurements were taken from distinct samples or whether the same sample was measured repeatedly
☐	☒	The statistical test(s) used AND whether they are one- or two-sided<br>*Only common tests should be described solely by name; describe more complex techniques in the Methods section.*
☒	☐	A description of all covariates tested
☒	☐	A description of any assumptions or corrections, such as tests of normality and adjustment for multiple comparisons
☐	☒	A full description of the statistics including central tendency (e.g. means) or other basic estimates (e.g. regression coefficient) AND variation (e.g. standard deviation) or associated estimates of uncertainty (e.g. confidence intervals)
☒	☐	For null hypothesis testing, the test statistic (e.g. *F*, *t*, *r*) with confidence intervals, effect sizes, degrees of freedom and *P* value noted<br>*Give P values as exact values whenever suitable.*
☒	☐	For Bayesian analysis, information on the choice of priors and Markov chain Monte Carlo settings
☒	☐	For hierarchical and complex designs, identification of the appropriate level for tests and full reporting of outcomes
☒	☐	Estimates of effect sizes (e.g. Cohen's *d*, Pearson's *r*), indicating how they were calculated
☒	☐	Clearly defined error bars<br>*State explicitly what error bars represent (e.g. SD, SE, CI)*

Our web collection on statistics for biologists may be useful.

Software and code

Policy information about availability of computer code

Data collection	N/A
Data analysis	PRISM 7.04 by GraphPad Software, Inc. FlowJo 10.4.2 by FlowJo, LLC.

For manuscripts utilizing custom algorithms or software that are central to the research but not yet described in published literature, software must be made available to editors/reviewers upon request. We strongly encourage code deposition in a community repository (e.g. GitHub). See the Nature Research guidelines for submitting code & software for further information.

Data

Policy information about availability of data

All manuscripts must include a data availability statement. This statement should provide the following information, where applicable:

- Accession codes, unique identifiers, or web links for publicly available datasets
- A list of figures that have associated raw data
- A description of any restrictions on data availability

The data that support the findings of this study are available from the corresponding author upon reasonable request.

Field-specific reporting

Please select the best fit for your research. If you are not sure, read the appropriate sections before making your selection.

☒ Life sciences ☐ Behavioural & social sciences ☐ Ecological, evolutionary & environmental sciences

For a reference copy of the document with all sections, see nature.com/authors/policies/ReportingSummary-flat.pdf

Life sciences study design

All studies must disclose on these points even when the disclosure is negative.

Sample size	Where applicable, the sample size for each study was selected to ensure each study was statistically well powered.
Data exclusions	No data was excluded.
Replication	All attempts at replication were successful.
Randomization	Randomization was not relevant for this study.
Blinding	The investigators performing the binding and neutralization assays were blinded.

Reporting for specific materials, systems and methods

Materials & experimental systems

n/a	Involved in the study
☐	☒ Unique biological materials
☐	☒ Antibodies
☐	☒ Eukaryotic cell lines
☒	☐ Palaeontology
☒	☐ Animals and other organisms
☐	☒ Human research participants

Methods

n/a	Involved in the study
☒	☐ ChIP-seq
☐	☒ Flow cytometry
☒	☐ MRI-based neuroimaging

Unique biological materials

Policy information about availability of materials

Obtaining unique materials	All materials are available from the authors without restriction for non-commercial use.

Antibodies

Antibodies used	Anti-human CD3 PerCP/Cy5.5, Biolegend, Cat #: 300430, Lot #: B251283, Clone: UCHT1; anti-human CD8 PerCP/Cy5.5, Biolegend, Cat #: 344710, Lot #: B238358, Clone: SK1; anti-human CD14 PerCP/Cy5.5, Thermo Fisher Scientific, Cat #: 45-0149-42, Lot #: 1933247, Clone: 61D3; anti-human CD16 PerCP/Cy5.5, Biolegend, Cat #: 302028, Lot #: B241057, Clone: 3G8; anti-human CD19 APC/Cy7, Biolegend, Cat #: 302218, Lot #: B234156, Clone: H1B19; anti-human CD20 APC/Cy7, Biolegend, Cat #: 302314, Lot #: B234522, Clone: 2H7; anti-human IgD BV510, BD Biosciences, Cat #: 563034, Lot #: 7193788, Clone: IA6-2; anti-human IgM PE/Cy7, Biolegend, Cat #: 314532, Lot #: B246228, Clone: MHM-88; anti-human IgA DyLight 488, abcam, Cat #: ab98553, Lot #: GR297260-3, Clone: goat polyclonal; anti-human IgG BV605, BD Biosciences, Cat #: 563246, Lot #: 7026520, Clone: G18-145; anti-human CD45RB FITC, Biolegend, cat # 310205, Clone: MEM55; Anti-human CD5, BD Biosciences, Cat #: 563945, Clone UCHT2.
Validation	All secondary antibodies were functionally validated prior to use.

Eukaryotic cell lines

Policy information about cell lines

Cell line source(s)	All cell lines were sourced from ATCC.
Authentication	All purchased cell lines were used as is from the supplier. No additional authentication was performed.
Mycoplasma contamination	The cell lines were not tested for mycoplasma contamination.
Commonly misidentified lines (See ICLAC register)	N/A

Human research participants

Policy information about studies involving human research participants

Population characteristics	Population characteristics are provided in Table S1 of the manuscript.
Recruitment	Study subjects aged 2.5 to 4 years of age were identified during their pre-operative visit to Otolaryngology, where an independent decision was made about the clinical indications for their tonsillectomy/adenoidectomy. Informed consent to participate in this study was obtained during the pre-operative visit.

Flow Cytometry

Plots

Confirm that:

- [x] The axis labels state the marker and fluorochrome used (e.g. CD4-FITC).
- [x] The axis scales are clearly visible. Include numbers along axes only for bottom left plot of group (a 'group' is an analysis of identical markers).
- [x] All plots are contour plots with outliers or pseudocolor plots.
- [x] A numerical value for number of cells or percentage (with statistics) is provided.

Methodology

Sample preparation	Heparinized blood (6-10 cc) was obtained from subjects at the time of surgery. Adenoid tissue removed during surgery was transferred to the laboratory for immediate processing. B cells were isolated from blood and adenoid tissue using a B cell enrichment column (Miltenyi MACS). Isolated B cells were stained with a panel of secondary antibodies (detailed above) and sorted by flow cytometry.
Instrument	BD FACSARIA fusion by BD Biosciences.
Software	FlowJo 10.4.2 by FlowJo, LLC.
Cell population abundance	RSV F-specific B cells were sorted in single-cell mode. The purity of the sort was confirmed by sequencing (i.e. each well only returned a single antibody sequence).
Gating strategy	The gating strategies used for B cell sorting are shown in Figure S1 and Figure S5.

- [x] Tick this box to confirm that a figure exemplifying the gating strategy is provided in the Supplementary Information.
